# Chitin-fibroin-hydroxyapatite membrane for guided bone regeneration: micro-computed tomography evaluation in a rat model

**DOI:** 10.1186/s40902-016-0060-6

**Published:** 2016-03-22

**Authors:** Young-jae Baek, Jung-Han Kim, Jae-Min Song, Sang-Yong Yoon, Hong-Sung Kim, Sang-Hun Shin

**Affiliations:** 1 Department of Oral and Maxillofacial Surgery, School of Dentistry, Pusan National University, Mulgeum-eup, Yangsan, 50612 Republic of Korea; 2 Department of Oral and Maxillofacial Surgery, Good Gang-An Hospital, Busan, 48256 Republic of Korea; 3 Department of Biomaterials Science, College of Natural Resources and Life Science, Pusan National University, Miryang, 50463 Republic of Korea

**Keywords:** Chitin-fibroin-hydroxyapatite, Guided bone regeneration, Micro-computed tomography, Rat calvarial defect

## Abstract

**Background:**

In guided bone regeneration (GBR) technique, many materials have been used for improving biological effectiveness by adding on membranes. The new membrane which was constructed with chitin-fibroin-hydroxyapatite (CNF/HAP) was compared with a collagen membrane (Bio-Gide®) by means of micro-computed tomography.

**Methods:**

Fifty-four rats were used in this study. A critical-sized (8 mm) bony defect was created in the calvaria with a trephine bur. The CNF/HAP membrane was prepared by thermally induced phase separation. In the experimental group (*n* = 18), the CNF/HAP membrane was used to cover the bony defect, and in the control group (*n* = 18), a resorbable collagen membrane (Bio-Gide®) was used. In the negative control group (*n* = 18), no membrane was used. In each group, six animals were euthanized at 2, 4, and 8 weeks after surgery. The specimens were analyzed using micro-CT.

**Results:**

Bone volume (BV) and bone mineral density (BMD) of the new bone showed significant difference between the negative control group and membrane groups (*P* < 0.05). However, between two membranes, the difference was not significant.

**Conclusions:**

The CNF/HAP membrane has significant effect on the new bone formation and has the potential to be applied for guided bone regeneration.

## Background

Guided bone regeneration (GBR) is a valuable procedure that uses a membrane to reconstruct bone defect in the oral and maxillofacial area [1]. As a barrier of bone defect, a membrane guards the region from invasion of in-growth cells from fibrous connective tissue and promotes a favorable environment for cells derived from bone marrows [2]. For achieving a competent result, the GBR barrier membrane requires certain properties such as bioactivity, biocompatibility, cell viability, and space-maintaining ability during the bone healing process [1].

Since first reported by Boyne [3], who used a cellulose acetate filter to regenerate bone in surgically created mandibular defect, numerous materials for the membrane have been introduced for enhancing the results of osteogenesis. A number of membranes are often divided based on degradability. Non-degradable membranes such as expanded polytetrafluoroethylene (ePTFE) are relatively thin and can maintain sufficient intensity during the bone healing process; however, it must be removed by a secondary operation, while degradable membranes do not require additional procedures [4, 5]. Nowadays, the use of resorbable membranes is increased by clinicians and the most widely used membrane is Bio-Gide® (Geistlich Pharma AG, Wolhusen, Switzerland), which is made of porcine collagen [6].

In our previous study, we reported the comparative study of a chitosan-fibroin-hydroxyapatite (CFB-HAP) membrane and collagen membrane for bone regeneration [7]. In that experiment, the CFB-HAP membrane showed significant potential as a guided bone regeneration membrane. However, Shi et al. [8] reported that chitosan has a peculiar ability of granulation tissue formation on dermo-epidermal lesions. And its stimulating effect of inflammatory cell which has an inhibitory effect of bacterial growth can lead to overexpressed inflammatory response in vivo [9, 10]. To solve this problem, many studies examined the application of chitin, acetylated chitosan, which arouse mild inflammatory response. In this study, a new membrane had been fabricated in which chitosan was replaced by chitin.

The purpose of this study is to compare the new bone formation and healing in rat skull defect using the new chitin-fibroin-hydroxyapatite membrane and collagen membrane (Bio-Gide®), which has been widely used at dental offices as a barrier membrane of guided bone regeneration, by means of computerized tomography (micro-CT) analysis.

## Methods

The material used in this study was a fabricated composite using chitosan, calcium hydroxyapatite, and fibroin. Acetylation reaction was carried out, and the composite was squeezed to obtain a membrane form [11].

### Fabrication of a membrane

Prepared for the creation of new membrane materials were the following: chitosan (Taehoon-bio Corp., Korea), hydroxyapatite nanopowder (Sigma-Aldrich Corp., USA), and raw silk (produced by *Bombyx mori* silkworms, degummed and dissolved in a mixed solvent of CaCl_2_, H_2_O, and ethanol; fibroin aqueous solution). Chitosan was purified with diluted 2 wt% aqueous acetic acid and 5 wt% sodium hydroxide solutions. The fibroin aqueous solution was dialyzed in flowing water for 7 days. The hydroxyapatite nanoparticles were treated in dry CO_2_ atmosphere for 48 h at 900 °C in electric furnace. With this process, the carbonate group was substituted with the hydroxyl group and/or the phosphate group of hydroxyapatite. The carbonate hydroxyapatite was thus synthesized. The chitosan/carbonate hydroxyapatite composite was prepared as follows. Carbonate hydroxyapatite was uniformly dispersed in 2 wt% acetic acid solution; 3 wt% chitosan was dissolved in carbonate hydroxyapatite-dispersed solution at room temperature. The composites were solidified in 10 wt% NaOH solution after casting on a glass plate. The fabricated composites of film type were washed with distilled water several times and were dried slowly at room temperature. The chitosan/carbonate hydroxyapatite composites reacted with 1 M acetic anhydride in methanol solution. The acetylation reaction was carried out with stirring at 120 rpm at 25 °C for 24 h. After the reaction, the composites were washed with methanol solution to remove unreacted acetic anhydride and by-products. The amino groups on the surface of the composites were substituted with the acetamide groups by acetylation.

### Animal models and surgical procedures

Fifty-four Sprague-Dawley albinic male rats (15 weeks old, Koatech, INC., Korea) weighing approximately 600 to 800 g were used as the animal experimental model in this study. The venue used for this study was the Laboratory Animal Resource Center of Pusan National University Yangsan Campus, under licensed by the Pusan National University Institutional Animal Care and Use Committee (PNU­2011­000241).

The animals were anesthetized with a mixture of 10 mg/kg of xylazine hydrochloride (Rumpun®, Bayer, Korea) and 100 mg/kg of ketamine chloride (Ketalar®, Yuhan Corporation, Korea). The dorsal area of rat cranium was shaved before the surgery, while the surgical field was prepared with an iodine solution. A midline incision was performed on the skin following the muscle and periosteum; the periosteum with muscles was reflected laterally. About 8-mm diameter of bony defect was then created in the center of the calvaria, with an 8-mm diameter trephine bur (Hee Sung Cor. Seoul, Korea). This defect is considered a critical-size bone defect because it does not heal spontaneously during the lifetime of the animal [12]. Each membrane was trimmed off to be rectangular (10 mm × 10 mm), enough to cover the bony defects, and then applied at the outer surface of the bony defect. The muscle layers were closed with 4-0 Vicryl® sutures in a continuous fashion, while the skin was closed with 3-0 Vicryl® sutures. Gentamycin 5 mg/kg was injected for the prevention of side effects after surgery. In the experimental group (*n* = 18), chitin-hydroxyapatite-fibroin membrane was used. In the control group (*n* = 18), an absorbable collagen membrane (Bio-Gide®) was used. In the negative control group (*n* = 18), a membrane was not used. In each group, six animals were euthanized by CO_2_ at 2, 4, and 8 weeks post-surgery. After euthanasia, skulls were harvested and fixed in 10 % formalin. They were taken to the micro-CT.

### Measurement of BV and BMD and 3D reconstruction using micro-CT

Micro-CT scans were performed with a SKYSCAN 1172 high-resolution micro-CT (SkyScan N. V., Belgium). Prior to scanning the specimens, a calibration scan was performed using a synthetic bone, water, and air sample. The exposure parameters were 70 kV and 140 μA. The total scan time was 20 min. The reconstruction of scanned images was done using the software (SkyScan CT-analyzer) after calibrating the bone, water, and air standard values. CT images were reconstructed using a modified cone-beam algorithm [13] with an isotropic voxel spacing of 0.027 × 0.027 × 0.027 mm^3^. The reconstructed three-dimensional (3D) image was then traced in three dimensions to the circumference of the original defect margins. This allowed the creation of a 3D reconstruction of the defect, referred to as the region of interests (ROI). The ROI of each specimen was analyzed for bone volume (BV) and bone mineral density (BMD).

### Statistical analysis

The bone defect was regarded as the statistical unit. Data were expressed as mean ± standard deviation (SD). One-way analysis of variance (ANOVA) was performed to analyze BV and BMD. Each group was compared using the Tukey post hoc test when a significant result was presented. The level of significance was set to 0.05.

## Results

### Gross examination

In the negative control group, there was no inflammatory change of tissue in all-sacrificial time. In the positive control (Bio-Gide®) group, after 2 weeks, the membrane was resorbed a little. After 4 and 8 weeks, the membranes were all resorbed and showed a good healing state. In the experimental (chitin-fibroin-hydroxyapatite (CNF/HAP)) group, the membranes were not resorbed until 8 weeks after surgery and mild inflammation was noted.

### Micro-CT analysis

#### Bone volume

**Fig. 1:**
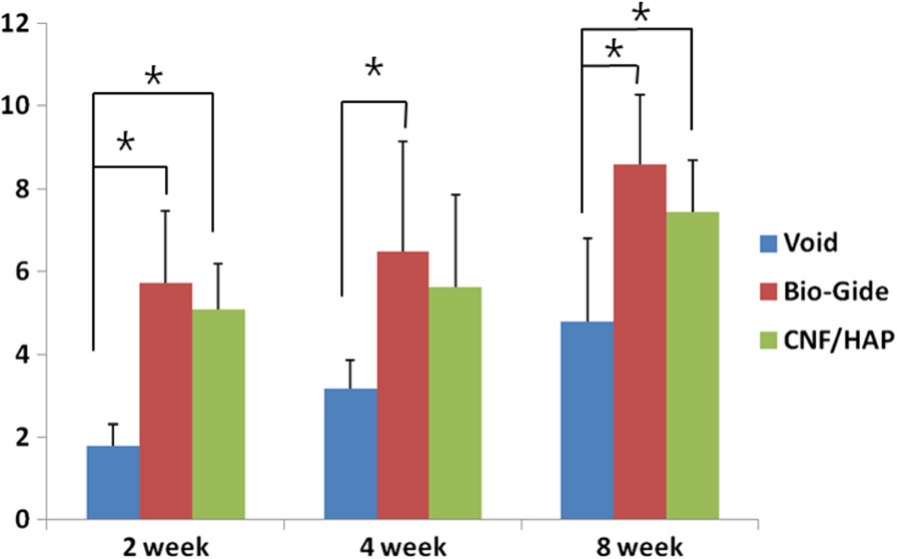
Measurements of bone volume by micro-CT analysis (mm^3^). *CNF/HAP=* chitin-fibroin-hydroxyapatite membrane

BV of the new bones that filled the bony defect showed significant difference between the negative control group and membrane groups, except the result of 4 weeks after surgery. However, showing comparison between the two membranes, the difference was not significant at the time of all observations, although the bone volume level of the Bio-Gide® group was higher than that of the CNF/HAP group (Table 1, Fig. 1).

**Table 1:** Measurements of the rat calvarial defect

Measurement	Time	Membrane			*P* value
		Void	Bio-gide®	CNF/HAP	
BV	2 weeks	1.79 ± 0.52^A^	5.73 ± 1.74^B^	5.08 ± 1.10^B^	0.001*
	4 weeks	3.16 ± 0.68^A^	6.49 ± 2.65^B^	5.63 ± 2.22^AB^	0.027*
	8 weeks	4.78 ± 2.03^A^	8.59 ± 1.66^B^	7.43 ± 1.28^B^	0.007*
BMD	2 weeks	0.45 ± 0.04^A^	0.62 ± 0.04^B^	0.59 ± 0.10^B^	0.029*
	4 weeks	0.58 ± 0.06^A^	0.79 ± 0.05^B^	0.67 ± 0.01^C^	0.019*
	8 weeks	0.67 ± 0.08	0.79 ± 0.04	0.76 ± 0.19	0.213

Different uppercase letters indicate statistical difference within the same line; *P < 0.05

*BV=* bone volume (mm^3^), *BMD=* bone mineral density (mg/ml), *CNF/HAP=* chitin-hydroxyapatite-fibroin membrane

#### Bone mineral density

**Fig. 2:**
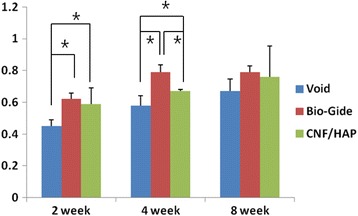
Measurements of bone mineral density by micro-CT analysis (mg/ml). *CNF/HAP=* chitin-fibroin-hydroxyapatite membrane

BMD measurements also showed significant yet different results compared with the negative control group and membrane groups. Similar to previous experimental measurements, a tendency was seen wherein the result of the membrane groups was higher than that of the negative control group. However, according to statistical tests, differences were observed in the period. In 2 weeks, the results showed a similar pattern in the bone volume; the postoperative 4 weeks showed significant differences between the Bio-Gide® group and experimental group. At 8 weeks after operation, no significant difference was detected among all groups including the uncovered group (Table 1, Fig. 2).

#### 3D reconstruction images

**Fig. 3:**
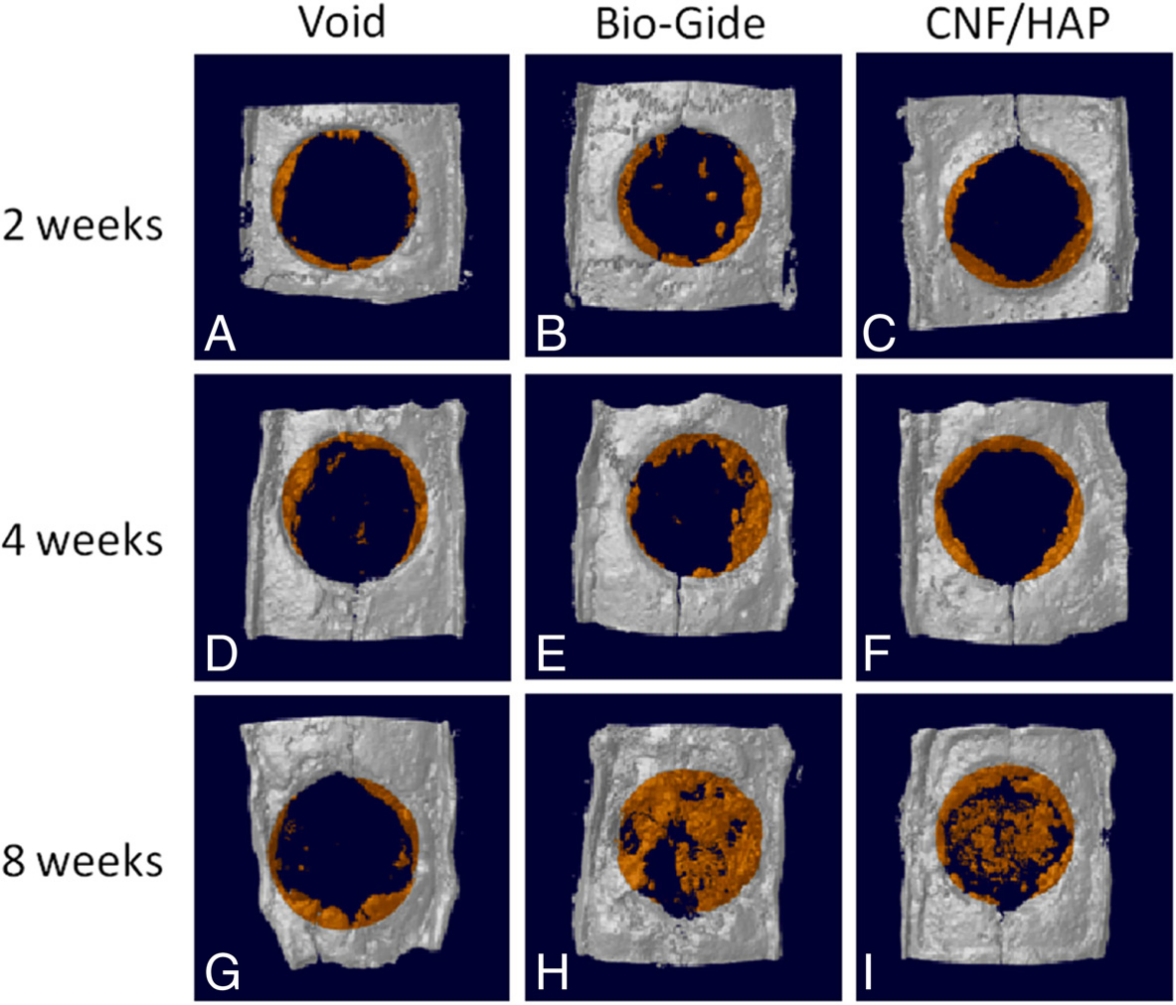
The 3D reconstruction images of each groups at 2, 4, and 8 weeks. **a** 2-week negative control group. **b** 2-week control group. **c** 2-week experimental group. **d** 4-week negative control group. **e** 4-week control group. **f** 4-week experimental group. **g** 8-week negative control group. **h** 8-week control group. **i** 8-week experimental group. The new bone formations were examined in the two membrane groups more than in the negative control group. Bone formation was increased in a time-dependent manner and could be detected through the 3D images

Figure 3 shows the 3D reconstruction images where a new bone was formed from the margin and the increase in dependent time. There were more new bones in the covered groups than in the void group.

## Discussion

In this study, chitin as a form of acetylated chitosan was used to resolve the problem of chitosan, inflammatory reaction. Chitosan has been promoting the effect of wound healing by stimulating inflammatory cell, and it has an inhibitory effect of bacterial growth which can lead to overexpressed inflammatory response in vivo [9, 10]. The main biochemical effects of chitosan are a fibroblast activation, cytokine production, and giant cell migration. Chitosan has a peculiar ability to foster adequate granulation tissue formation accompanied by angiogenesis and regular deposition of thin collagen fibers, a property that further enhances correct repair of dermo-epidermal lesions [8]. To solve this problem, many studies examined the application of chitin, which arouses mild inflammatory response. Chitin is a polyheterosaccharide comprised of glucosamine and *N*-acetyglucosamine units linked by 1-4 glucosidic bonds; it has the properties of biodegradability, biocompatibility, chemical inertness, wound healing, and antibacterial and anti-inflammatory activities [14]. Hence, chitin has recently gained interest for its use in GBR membranes, as many studies present positive results [15–18]. However, despite these attempts to decrease or resolve the inflammatory reaction, somewhat sight inflammations were observed in the experiment on animals of this study. 

Despite good results from many studies which used chitin as a membrane for guided bone regeneration [15–18], problems with the exclusive use of chitin involve its poor mechanical properties. Addition of hydroxyapatite improves the mechanical properties of chitin [19]. However, this is a secondary objective; the main purpose using hydroxyapatite in this study is to enhance the result accompanying the ability of osteoconductivity. Hydroxyapatite, which is a natural inorganic component of the bone and teeth, has already been used in orthopedics and dentistry because of its osteoconductivity and osteophilicity [20, 21], and it is used usually in particulate form as a scaffold. Polymers combined with hydroxyapatite are capable of promoting osteoblast adhesion, migration, differentiation, and proliferation, especially useful for potential applications in bone repair and regeneration [22, 23]. Hydroxyapatite particles have been incorporated into chitosan matrixes to enhance the bioactivity of tissue-engineering scaffolds for hard tissue regeneration [24].

The GBR barriers should have appropriate mechanical properties such as sufficient strength and elasticity to cover bone defects and block a down-growth of surrounding connective tissues in the implantation region. Silk fibroin acts as an enzyme immobilization matrix with good mechanical properties, as it has blood compatibility and good dissolved oxygen permeability [25, 26]. Reports have been made on silk fibroin/chitosan membranes with good mechanical properties forming an interpenetrating polymer network [27–29].

Bio-Gide® (Geistlich AG, Wolhusen, Switzerland), which is composed of porcine type I and type III collagen fibers, and has a bilayer structure composed of a “compact” and “porous” layer, is a well-known and commonly used collagen membrane for guided bone regeneration as an absorbable membrane [30]. Membrane-derived collagen fibers may trap some osteoinductive factors such as bone morphogenetic proteins, transforming growth factors, insulin-like growth factors, and fibroblast growth factors that can be easily released from the bone matrix when the artificial bone cavity was prepared. Even if collagen itself has no ability to bind these osteoinductive factors, it can bind several extracellular matrixes that have high affinity for these factors [31]. If so, several osteoinductive factors may be possibly trapped by collagen fibers of the membrane and make previously migrated cells differentiate into an osteoblastic lineage. In addition, type I collagen may directly play an important role in the osteoblastic differentiation [32]. For the aforementioned reasons, Bio-Gide® has been used by many clinicians as it gained a reported high success rate when used for GBR.

The hypothesis in this experiment was as follows. When the chitin-hydroxyapatite-fibroin composite is applied as a membrane for guided bone regeneration, the relatively fast degradation of chitin makes room for the growth of new tissue and increases the opportunity to assimilate hydroxyapatite with the host bone [33]. However, an unabsorbed membrane remained at 8 weeks in the experimental group. This might have been due to containing hydroxyapatite. Although hydroxyapatite is a bioresorbable material, sufficient time for complete absorption is required. According to the report of Jansen et al. [34], depending on the ratio of the hydroxyapatite, at least 4 weeks is required for the identification of gross absorption. At 8 weeks of the experimental group, the time interval might be the cause of the remnant of membranes. The time interval which was set in this study was the result of considering clinical application, where the time intervals of about 6 weeks or longer between the operation and secondary surgery or implantation [35] are generally applied for guided bone regeneration. It might be carefully expected that this problem could be solved, if it is applied for a longer period.

Overall, the covered groups showed excellent results compared with the uncovered group. However, based on the comparative period, some difference was indicated in the comparison of bone volume. These results can be related to the experimental design. This study did not use a filler to compare only the pure effect of the membranes. Because of this, it might have varying degrees of difference of the results. Schmid et al. also reported certain variations of bone healing when using bioresorbable membranes without filler materials [36]. Although no statistical differences showed in bone volume at 4 weeks between the void group and experimental group, the overall results do not deviate from the flow. A similar result appears in BMD measurement. At 4 weeks after surgery, there are higher values in the Bio-Gide^Ⓡ^ group than in the new membrane group in terms of BMD measurement. However, 8 weeks after surgery, these differences disappeared, which shows similar results with the control group. This might be due to the aforementioned experimental design, if only limited to the result of 4 weeks. However, it would be reasonable to infer the cause to consider the characteristic of the BMD which is a measurement of the bone maturity and could be affected by an inflammatory response. If so, the results which showed the not significant difference of BMD after 8 weeks, regardless of the amount of the regenerated bone of all groups, could be interpreted in the same point of view.

## Conclusions

In this study, the CNF/HAP in the form of a membrane was applied as the barrier membrane for guided bone regeneration in a rat model. To assess the effectiveness, micro-CT analysis and visualizations of the regenerated osseous tissue with 3D reconstruction program were used. In the positive control and experimental group, the bone volume and bone mineral density, which are indexes of bone regeneration, have higher values compared with those in the negative control group. Similar results were indicated comparing two membranes that had been used in this report.

1. The CNF/HAP has a similar bone regeneration ability compared with the collagen membrane.
2. The possibility of the CNF/HAP membrane is seen as a barrier membrane in GBR.
